# Distribution of water phase near the poles of the Moon from gravity aspects

**DOI:** 10.1038/s41598-022-08305-x

**Published:** 2022-03-16

**Authors:** Gunther Kletetschka, Jaroslav Klokočník, Nicholas Hasson, Jan Kostelecký, Aleš Bezděk, Kurosh Karimi

**Affiliations:** 1 Institute of Hydrogeology, Engineering Geology and Applied Geophysics, Faculty of Science, Charles University, 12843 Prague, Czech Republic; 2 Geophysical Institute, University of Alaska-Fairbanks, 903 N Koyukuk Drive, Fairbanks, AK 99709 USA; 3 Astronomical Institute, Czech Academy of Sciences, Fričova 298, 251 65 Ondřejov, Czech Republic; 4 Water and Environmental Research Center, Institute of Northern Engineering, University of Alaska Fairbanks, 1764 Tanana Loop, Fairbanks, AK 99775 USA; 5 Research Institute of Geodesy, Topography and Cartography, 250 66 Zdiby 98, Czech Republic; 6 Faculty of Mining and Geology, VSB-TU Ostrava, 708 33 Ostrava, Czech Republic; 7 Faculty of Civil Engineering, Czech Technical University in Prague, 166 29 Praha 6, Czech Republic

**Keywords:** Environmental sciences, Planetary science, Space physics, Astronomy and planetary science, Energy science and technology

## Abstract

Our Moon periodically moves through the magnetic tail of the Earth that contains terrestrial ions of hydrogen and oxygen. A possible density contrast might have been discovered that could be consistent with the presence of water phase of potential terrestrial origin. Using novel gravity aspects (descriptors) derived from harmonic potential coefficients of gravity field of the Moon, we discovered gravity strike angle anomalies that point to water phase locations in the polar regions of the Moon. Our analysis suggests that impact cratering processes were responsible for specific pore space network that were subsequently filled with the water phase filling volumes of permafrost in the lunar subsurface. In this work, we suggest the accumulation of up to ~ 3000 km^3^ of terrestrial water phase (Earth’s atmospheric escape) now filling the pore spaced regolith, portion of which is distributed along impact zones of the polar regions of the Moon. These unique locations serve as potential resource utilization sites for future landing exploration and habitats (e.g., NASA Artemis Plan objectives).

## Introduction

NASA’s return to the lunar surface (i.e. Artemis Plan) requires mission planning for near surface water phase resources^1^. The lunar surface regolith is coupled with ion-magnetohydrodynamical processes that may have contributed to the deposition of water phase on its surface. This is because the lunar environment is exposed for five days of each Earth orbit period to a magnetic field tail extending all the way from the Earth’s geomagnetic field^2^. Recent measurements from the Kaguya lunar orbiter (JAXA) have revealed significant numbers of oxygen ions during the time when the lunar orbit was inside the geomagnetic field^2^. This provided the necessary evidence that oxygen ions were not coming from the intrinsic solar winds. This is because the high temperature of the solar corona allows for only multi-charged oxygen ions (O5+, O6+, O7+ and O8+) as observed by Advanced Composition Explorer (ACE) space satellite with O+ in negligible amounts^3^. However, 1–10 keV O+ ions were observed to populate the Moon’s environment during the transition through the plasma sheet that originated from the Earth’s ionosphere^2,4^. The terrestrial ion’s flux density during the Moon’s passage through the magnetotail was estimated between 2.1 × 10^4^ cm^2^ s^−1^ and 2.6 × 10^4^ cm^−2^ s^−1 ^^2,5^.

This process of Earth-lunar geoionic O+ accumulation fluctuated over the history of the earth for several reasons: Initially (1), when the geomagnetic field may have not been well developed or even absent in ancient times^6^ the O+ accumulation was more intense. When applying this possibility from the Earth to Mars, where the global magnetic field is absent^7,8^, there, an ionospheric plasma sheet develops in absence of the global magnetic field and transfers ion, mostly oxygen, down the plasma sheet as observed by Mars Atmosphere and Volatile Evolution (MAVEN) spacecraft^9^. The second source (2) of ion enhancement has to do with the increasing distance between the Earth and the Moon in their history^10^. The third source (3) of ion variability is an episodical increase of the solar activity observed by MAVEN mission^11^. Evidence of such ion transfer mechanisms^9^ supports a hypothesis that a part of the terrestrial atmosphere that was lost in the past is now likely preserved within the surface of lunar polar regolith. For this hypothesis there is a support from observation of nitrogen and noble gases isotopes^12^. The recent advancements in Earth's atmospheric escape warrants new analysis of water phase deposits on the Moon. We apply our novel gravity aspects (descriptors) derived from harmonic potential coefficients of gravity field of the Moon by considering these novel ionic transfer mechanisms on depositional history of water phase formation, from which gravity strike anomalies appear.

**Figure 1:**
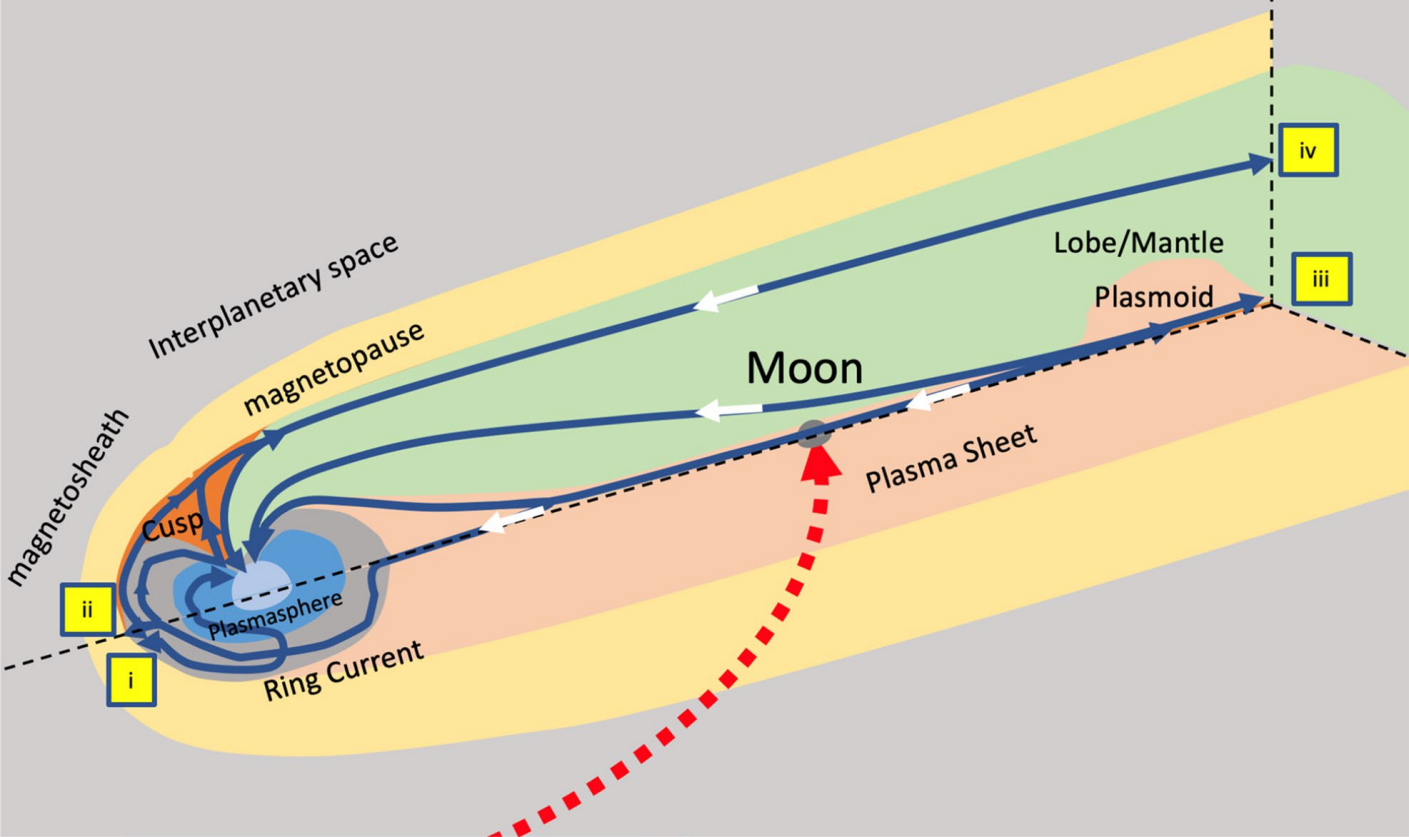
Sketch showing three-dimensional cutaway of Earth’s magnetosphere. The blue and white arrows are motion pathways of ions (for details see Seki et al.^5^) illustrating the mechanism for oxygen/hydrogen ions transfer to the Moon. Red dotted line with the arrow shows motion of the Moon into the magnetospheric tail. Escape locations into the interplanetary space is marked by locations i, ii, iii, iv. Image was drown using Microsoft PowerPoint for Mac Version 16.55.

While the loss of the ions from the atmospheres of terrestrial planets depends on processes at the atmosphere-surface interface, there are a significant loss mechanisms occurring in the upper atmosphere. For example, the ionosphere’s loss of ions due to space plasma acceleration can dynamically control the evolution of the atmosphere^5^. The geomagnetic field creates an obstacle to the solar wind preventing a direct abrasion of terrestrial neutral ions (oxygens, hydrogens) via thermal and non-thermal activities^13^. Four main pathways of terrestrial ions constitute of (i) magnetopause escape, (ii) magnetopause ring current dayside escape, (iii) anti-sunward flow escape, and (iv) lobe/mantle escape (Fig. 1). When ions are escaping via these pathways^5,14,15^, they can be returned towards the Earth and be added back to the atmosphere^5^. This occurs when the collision-less path distance becomes small enough that plasma on this length scale dissipates and the geomagnetic field lines and plasma field lines become reconnected^16^. Independently, ion outflow (including mostly H+, O+) from the uppermost terrestrial polar ionosphere has a time dependent typical thermal energies of 0.3 eV^16^. This terrestrial polar wind outflow is on the order of 10^25^ ions s^−1^ during the solar maximum activity, having average flow rates across the solar minimum and maximum of ~ 5 × 10^24^ ions s^−1^, caused by electric field disturbance due to charge difference between the ions and faster moving electrons^16^. Prior work has shown, during the dark sky conditions of lunar eclipses, that the differential effects of these energetic H+/O+ ionic species strike the Moon’s surface when engulfed by magnetotail transit. This observation suggested that omnipresent exospheric sources are augmented by these variable plasma impact sources in the solar wind reconnection with Earth’s magnetotail^17^. There is one order of magnitude difference between the estimates of the polar outflow and the four ion escape routes^5^. Here we consider this unknown loss of ions may account for volume of ions deposited on the Moon.

We note that the solar wind plasma separation of the electrons from the heavy ions is possible when the neutral plasma is obscured by an airless obstacle (asteroids, the whole Moon in this case)^18^. Plasma expands into the void behind the obstacle and creates an electron rich (ion free) volume behind the obstacle^19^. This mechanism was considered for the Lunar dust levitation^20^. The electrons are lighter and therefore they diffuse more efficiently into the shadow behind the Moon while the heavier positive ions continue further distances along the Moon’s shadow boundary (an analogy with the electrostatic signature present at permanently shadowed craters^18^). Note that laboratory experiments tried to model electrostatic accumulation due to shadow obstacle processes^21^. They considered shadows as a cause of an electrostatic lofting of the dust on airless bodies, the process that plays role in surface evolution. This may well relate to unexplained observations of dust ponding on asteroids. These dust ponds are accumulation of dust formed in craters on 433 EROS^22–24^. Similar observations were made on comet 67P^25^. Even at Saturn's icy moon Atlas, where the unusually smooth surface may have been modified by electrostatic field. Thus, there must be a distance at which the Moon’s electrostatically charged tail may contribute to the reconnection events in the Earth’s geomagnetic tail (Fig. S10). Each time when the electrostatically charged tail from the Moon enters the geomagnetic plasma sheet, it may interfere with any ions present in the plasma sheet and modify their trajectories. Such disturbance in particle motion may result in the collision-less path distance of the plasma becoming significantly smaller and this could significantly increase the probability of magnetic flux lines’ reconnection.

Earth's atmospheric escape warrants important considerations as potential life support pathways on the Moon^26–28^. We calculate a rough estimation of volumetric water phase that has likely deposited and transformed the Moon’s regolith over millennia: during the intersecting 5-day interaction of Earth’s magnetosphere with the Moon, if we assume only 1% of the average ion flow per second (5 × 10^24^ ions s^−1^) of the O+/water molecules are deposited into the Moon’s regolith, this volumetric time transfer equates to 1 million × 24 h × 5 days × 12 months × 5 × 10^22^ water molecules × 2.7^–29^ m^3^/millions of years (MY) is ~ 1 km^3^ per MY (This calculation assumed 0.3 nm size of the water molecule, thus 0.027 nm^3^ = 2.7^–29^ m^3^ water phase volume). If we assume this process occurring from the period of Late Large Bombardment ~ 3.5 billion years (BY) ago, based on the above calculations, we estimate the accumulation of ~ 3500 km^3^ of terrestrial water phase, filling the pore spaced regolith, for which novel gravity strike signals would appear. This amount may be two to three fold different, due to smaller Moon-Earth distance in the history^10^, but not different by more than order of magnitude. For example, this volume, of ~ 3500 km^3^ would be similar to the ~ 5400 km^3^ volume of lake Vostok in Antarctica^29^.

Ionic flow is orientated both away from and towards the Earth, due to energetic escape processes of Earth's atmosphere. When the Moon enters and is exposed to Earth’s ionic plasma sheet, this may capture ions and account for the missing portion of the ionic budget^5^. Impact gardening would then distribute these deposits across the whole Moon’s surface. Most primitive basalts from the lunar surface contain considerable amounts of H_2_O^30,31^. However, the solar radiation would evaporate the surface deposits and redistribute them towards the polar regions^32,33^. Larger amounts of such deposits would form a permafrost at the near surface polar regions of the Moon, while filling the pore space of the lunar regolith, and over time, compressing into the liquid phase boundary at depth^34^. Based on the pressure variation with depth on the Moon, we get into 1 atmosphere regolith overpressure at depth of 30 m^35^. The temperature near the Moon’s poles is about 100 K and the regolith there has an increasing thermal gradient with depth of about 0.1–0.5 K/m^36^. From this gradient we estimate a depth between 100 and 2000 m, where the pressure and temperature would allow water in pores to exist in liquid state. We have a prior experience with detecting subsurface water phase deposits on Earth. For that detection, we used gravity aspects and estimate potential locations of underground deposits of water phase and gas in Sahara Desert regions^37^. Here we apply these methods and locate potential deposits of water phase in the polar regions of the Moon.

## Methodological theory on data

We use a novel method for detecting underground density anomalies via anomalous gravity signal. This method was developed for the study of various geological structures on the Earth: impact craters, subglacial volcanoes, lake basins, paleolakes or petroleum deposition sites globally. Notable, this has also been extended for the impact craters, maria and catenae on the Moon^38,39^. Typical gravity investigations employ the traditional gravity anomalies or second radial derivatives of the disturbing gravitational potential. This work uses a wider set of functions of the disturbing gravitational potential, we call them “*gravity aspects*”. These are derivation operators acting on the *gravity anomalies Δg*, the *Marussi tensor* (**Γ**)**,** the second derivatives of the disturbing potential (*T*_*ij*_), with the second radial component *T*_*zz*_, two of the three *gravity invariants* (*I*_*j*_), their *specific ratio* (*I*), the *strike angles* (*θ*) and the *virtual deformations* (*vd*). Our prior use revealed their diverse sensitivity to the underground density contrasts were due to causative bodies: these are computed to a high degree and order with sufficient numerical stability. It appears that such application extracts a finer and more complete detail of satellite gravity measurements. Theory of this approach was outlined in the book of Klokočník et al.^39^. Further examples and specific application of this method to the Moon is in the Supplementary material.

From Eq. (1) (see Supplementary material) we compute and plot the strike angles *θ* at the location of interest (here at the Moon’s polar regions). Alignment indicates the aligned porosity, filled with contrasting density material (water phase/vacuum). The aligned *θ* regions suggest water phase deposits.

The first step of this detection method is a transformation: we use the difference in the gravity anomalies between its assumed deepest and shallowest location, then, to the difference in the vertical direction, allowing the maximum estimate where the object can be located, and how large/deep it might be. Several iterations are required to achieve this step. The second step is a use a topography data and the geographic positions of topographical sites, leading eventually to a fine-tuning of the level, extent, and shape of the water phase enriched objects.

### Gravity data

The input data here uses harmonic potential coefficients of the spherical harmonic expansion to degree and order d/o of the perturbational gravitational potential (Stokes parameters). A set of these coefficients defines a global static gravitational field. We use the best models available based on satellite records^40,41^. This defines the limits of d/o = 1200 and 1500 for the models GRGM1200A^40^ and GL1500E^41^ respectively, with practically useful limit d/o = 600 (recommended by the authors of these models themselves). Application of these models allows for the theoretical ground resolution ~ 10 km. The precision is about 10 mGal. For this paper we chose the GRGM1200A model (after tests concerning degradation of gravity aspects for different harmonic degree, order, and/or appearance of any artifacts).

### Surface topography data

These are taken from a new lunar digital elevation model from measurements of the LOLA (Lunar Orbiter Laser Altimeter), an instrument on the payload of Lunar Reconnaissance Orbiter (LRO) spacecraft^42,43^. The height is given relative to the Moon’s reference radius of 1737.4 km. A nominal precision of the LOLA altimeter is ~ 10 cm.

## Results

**Figure 2:**
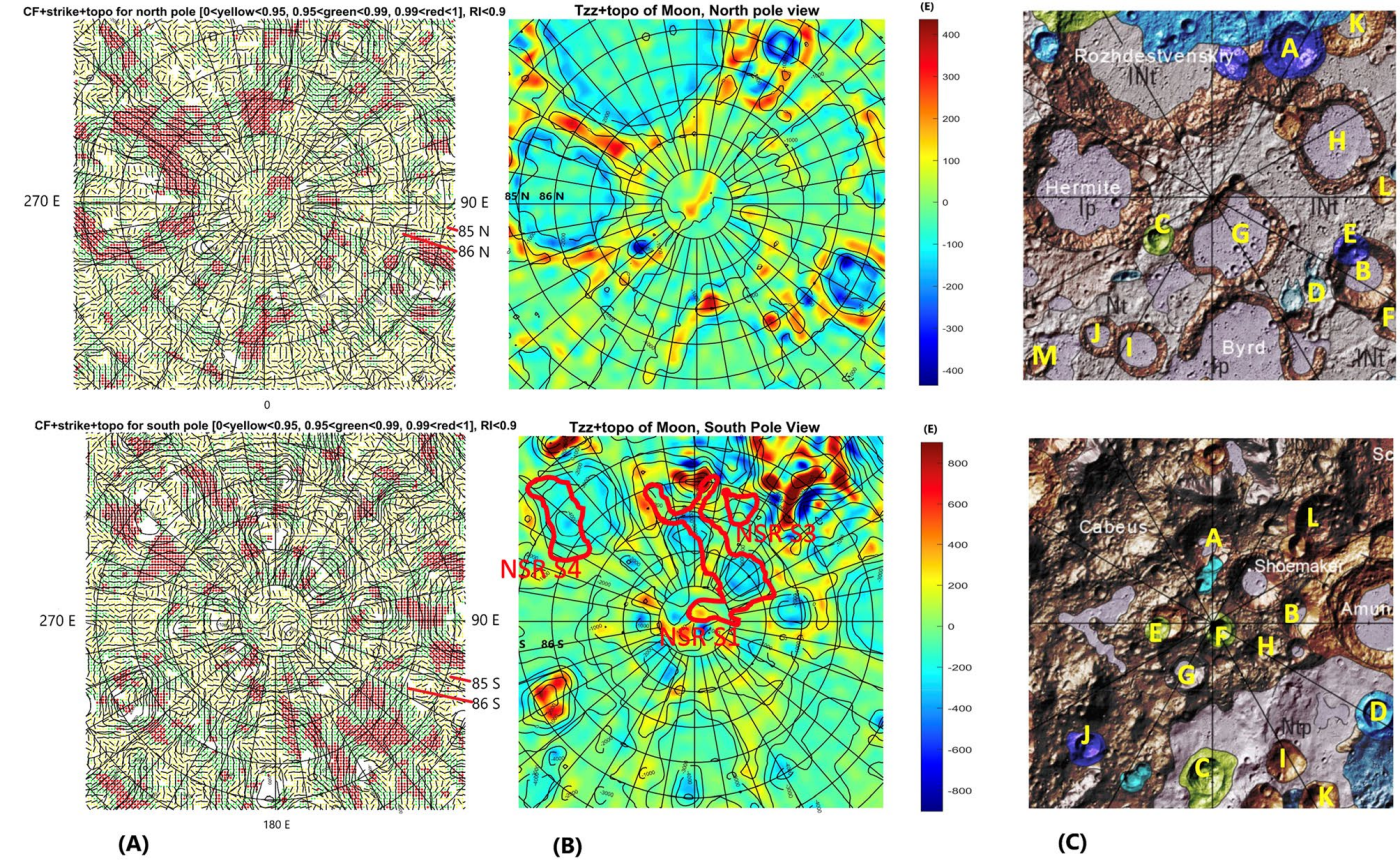
Geophysics, topography, and geological subunits of the Moons polar regions. (**A**) Gravity comb factor (CF) plotted for ratio *I* < 0.9 (see Eq. (2) and definition of CF in Supplementary information). The color legend identifies degree of alignment of strike angles (also Figs. 3, 4); (**B**) Gravity second derivative *T*_*zz*_ [E] along with topography [m]. Three areas outlined by red lines and labelled with NSR S1, NSR S3, and NSR S4 are regions identified with a potential water rich permafrost based on neutron suppression observations^34^; (**C**) Geological map units, where in north pole panel significant craters are labelled by yellow letters as: A—Rozhdestvenskiy U, B—Nansen F, C—Hermite A, D—Porges A, E—Porges B, F—Nansen C, G—Peary, H—Rozhdestvenskiy W, I—Porges C, J—Porges D, K—Porges E, L—Porges F, M—McCoy A, and craters in south pole panel as: A—Haworth, B—Faustini, C—Wiechert J, D—Idel’son L, E—DeGerlache, F—Shackleton, G—Sverdrup, H—Slater, I—Wiechert P, J—Kocher, K—Wiechert U, and L—Nobile; Data in plots (**A**), (**B**) were produced by combination of MATLAB, Surfer7.0 and Microsoft PowerPoint. (**C**) is a PowerPoint-modified Unified Geology Map of the Moon^44^.

**Figure 3:**
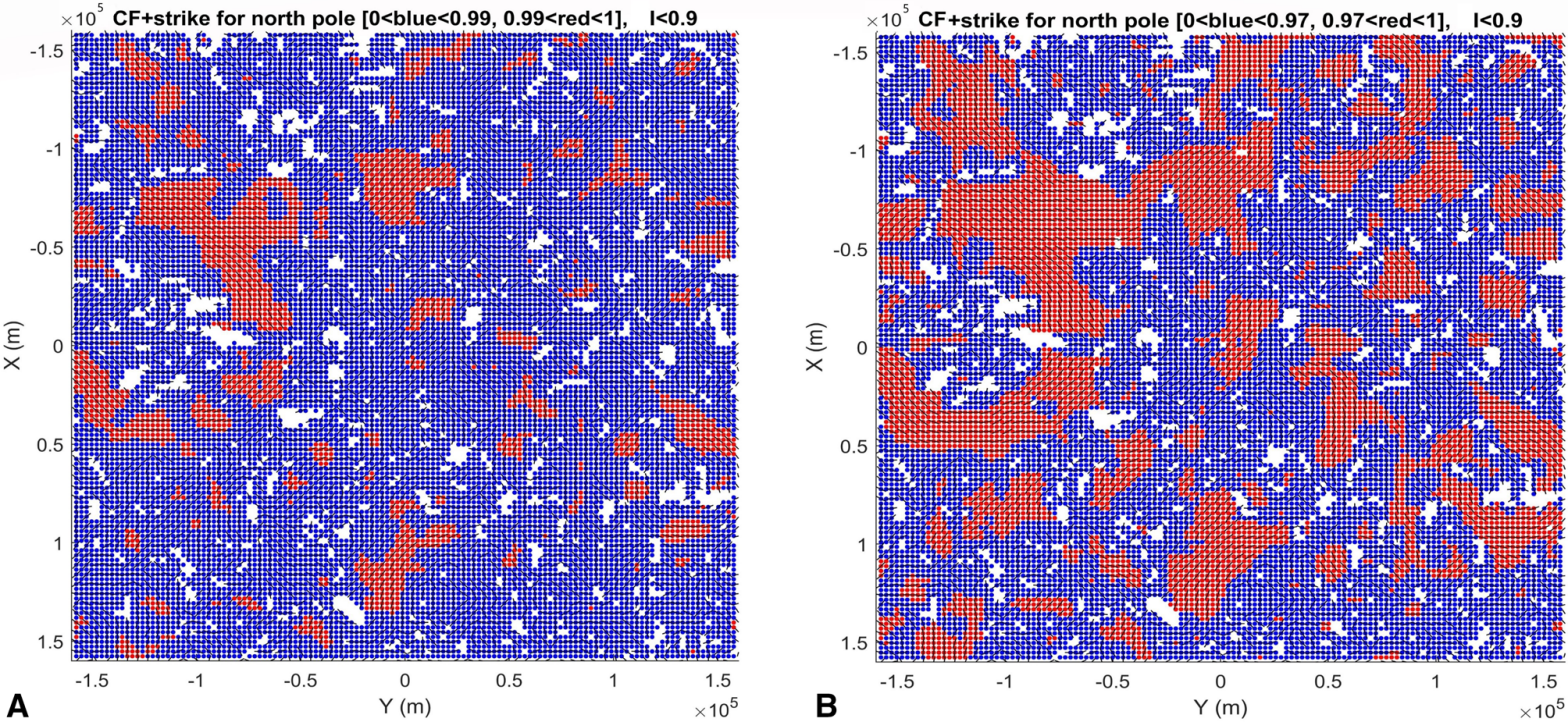
Stability of the comb factor (CF) within the area of north pole of the Moon. Dimensions are in meters. (**A**) Left panel corresponds to the strike angle plot and its CF for the north pole in Fig. 2A. The CF between 0.99 and 1.00 is in red color while the lower CF is in blue color. (**B**) Right panel shows CF between 0.97 and 1.00 in red color while the lower CF is in blue (blue symbol is larger for contrast clarity). Both plots are strike angles for ratio *I* < 0.9 (see Eq. 2), sensitive to weakness directions of the rocks in subsurface structures near the north pole of the Moon. Data were plotted using MATLAB software.

**Figure 4:**
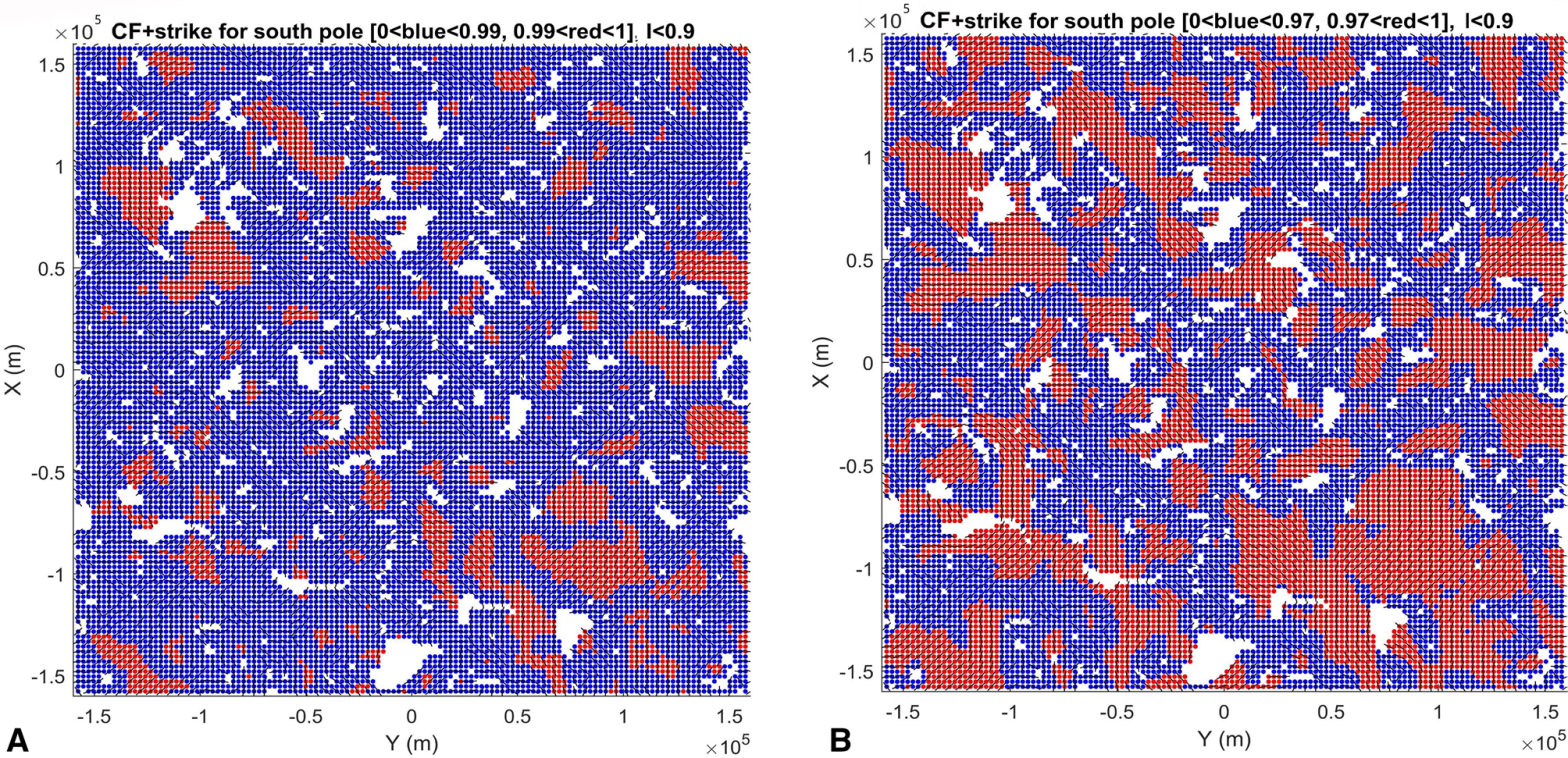
Stability of the comb factor (CF) within the area of south pole of the Moon. Dimensions are in meters. (**A**) Left panel corresponds to the strike angle plot and its CF for the south pole in Fig. 2A. The CF between 0.99 and 1.00 is in red color while the lower CF is in blue color. (**B**) Right panel shows CF between 0.97 and 1.00 in red color while the lower CF is in blue (blue symbol is larger for contrast clarity). Black letter P shows significant extent of CF-detected pores in the areas of Aitkin basin. Both plots are strike angles for ratio *I* < 0.9 (see Eq. 2), sensitive to weakness directions of the rocks in subsurface structures near the north pole of the Moon. Data were plotted using MATLAB software.

We computed and plotted the gravity aspects, namely the strike angles *θ* and the second radial derivatives *T*_*zz*_ near the lunar poles—see Fig. 2A,B and Supplementary material. In Fig. 2A, we used three color modes to express the degree of alignment: yellow and greed as misaligned and red with high degree of alignment. The choice of contrasting alignments (i.e., aligned vs non-alignment), was chosen to be the most conservative, so that only areas with high Comb Factor (CF) values (0.99–1.00) were shown in Fig. 2A (see Supplement for CF definition). To demonstrate the robustness, we show more than one way of plotting these strike angle parameters, and represented by CF (Figs. 3, and 4, and Supplementary material). This shows how we outline areas for smaller alignment of strike angles (CF < 0.97) for each respective hemisphere. Figures S1–S8 show variations of strike angles for both polar regions of the Moon and for ratio *I* < 0.3 (representing 2D-like structures) and *I* < 0.9 (3D-like structures). The calculations resulted in areas of high degree alignment of CF. Hence, we outlined these areas by red vs green and yellow symbols in Fig. 2A. Note areas of significant alignment of the strike angles near the north and south poles of the Moon (Fig. 2A).

Note that Fig. 2B shows how the *T*_*zz*_, second derivative of the disturbing gravitational potential distributes near the polar regions of the Moon. The values are spread between − 300 E to 300 E near north pole and from − 600 E to 1100 E near south pole. In the north pole region, the low values are indicative of compressional regime; thus, near surface rocks are denser and spread near the inner ring of the two large impact structures in upper left corner of the Fig. 2B (upper north pole panel). Note, the minimum values of *T*_*zz*_ reside inside smaller impact craters are expressed both in topographic and the geological unit mapping (Fig. 2B,C, upper panel). While we observe these three craters (e.g., Rozhdestvenskiy, Hermite, and Bird), we find these topographic and geological units map to share similarities; the *T*_*zz*_ parameter shows that Byrd crater has missing low values within its inner rim structure (see Fig. S9 for delta g). This may relate to a larger difference in *delta g* indicating a variation in compression force inside the craters. The Bird crater is more gravity equilibrated than the Rozhdestvenskiy and Hermile craters. The large topographical relief also seems to generate low values in *T*_*zz*_ in other three smaller craters, labeled as A, B., and C. in the north pole geology map. To check the deeper extent of these impact structures we compare *T*_*zz*_ with *∆g* (Fig. 2 and Fig. S9) and see much larger contrast in *∆g* for Rozhdestvenskiy and B craters, followed by Hermite crater. Positive topographic relief shows consistently larger values in *∆g* value. Similarly, we obtained values for *T*_*zz*_ and *∆g* near the south pole (Fig. 2B, lower panel and Fig. S9). Note a larger span of *T*_*zz*_ values and association of *T*_*zz*_ minima with the interior of impact structures and topographic heights with the positive *T*_*zz*_ values (Fig. 2B).

## Discussion

The strike angles *θ* derived in this work show sensitivity to the rock’s anisotropy^17–20^. These fractured rocks’ weakness and corresponding anisotropy, point to the directions of the strike angle *θ* and thus towards likely locations of volatile phases accumulations, including water phase*.* Our results in Fig. 2 shows patchiness of the locations where water phase may have accumulated and this is consistent with the recent molecular water detection by SOFIA^45^, where they observed at 6 μm emission feature at high lunar latitudes interpreted as patchy water phase enrichment, and as much as 100–400 μg/g of regolith^45^.

The Moon’s surface has been significantly modified with impact craters, energetical processes triggering structural extensions during the conversion of high impact kinetic energy into heat. The resulting explosive fractures splinter these regolith rock units and creates topographical indentation, producing a gravitational instability. The rocks in crater vicinity were dominantly compressed in a direction away from the crater center^46^. Such compression creates elongation and fracturing in perpendicular direction (parallel to crater perimeter)^47–49^. Many thrust faults are positioned in the subsurface near the perimeter of the crater, due to the impact energy forcing to remove the significant volume material from the inside of the crater^50^. With age, post craters collapse removes or diminish these topographical and gravitational reliefs^51^. The post crater collapse orientation (towards the middle) creates and magnifies the network of faults that are parallel to craters’ perimeters^51^. Thus, both impact and post impact processes enhance the anisotropy of rocks along the perimeter of the crater, by forming networks of fault system containing planar weaknesses that include planar pores, oriented along the perimeter direction. On Earth, these pore-spaces (i.e., porosity) often become filled with fluids as water and/or oils. Similarly, we apply the gravity expression of the planar weaknesses of the Moon’s impact craters. For this goal we apply a method of gravity detection of the planar network of weaknesses above the preexisting water-filled basins, which has allowed identification of paleolakes on Earth, now arid regions^37,52^. It appears that paleolakes hidden under thick layers in the Great Sand Sea of Western and Southern Egypt generate a special gravity aspect signature that we interpret to be related to the structural anisotropy of the sediment basin^37,53^. Here we apply the same approach with the hypothesis that the structural weakness of impact craters can be recognized in the gravity aspects. In addition, the gravity aspect, namely the strike angle(s), can determine, where the pore space is likely to be filled with significant amounts of water phase. Once the fractures are filled with water phase, that is more mobile, compared with the host rock system, fractures become subjects of significant pore forces and subsequent anisotropy of the stress field detectable from the gravity potential aspects^53^.

The Moons polar regions contain significant amount of water phase^33,34^. Our estimation (in the Introduction) when considering the forementioned reasonings, allow for theoretical calculations exceeding several thousand cubic kilometers of water phase. Such volume estimates require water phase enclosed in the pore containing rock units in the polar regions, which may cause structural extensions and fracturing. The network of pore fractures surrounding these impact craters is likely to develop due to regular impact crater structural degradation processes^54^ and thus would be the most reasonable location for water phase deposits in the polar regions of the Moon. The aligned *θ* regions tend to be near the impact craters and the angles *θ* are parallel to craters’ perimeters. For example, near the north pole we identified several highly aligned regions along the perimeter of the Rozhdestvenskiy crater and several such areas around the Hermite crater. This is a significant indication, that these two craters contain significant pore space structures that weaken the rock underneath the surface and create gravity strike anomalies. The identified regions of highly aligned strike angles (Fig. 2A,B) are thus likely to contain a significant amount of pores-filled water-phase at the subsurface pressure depths, and solid phase near surface (e.g. permafrost).

We observe similar analogies of the aligned strike angle detection of the water phase-filled pore space in rocks near the south pole of the Moon. Our invented Comb Factor (CF) parameter has anomalous values around the crater perimeters (Fig. 2A), pinpointing a significant potential for presence of the significant volume of pore space filled with water phase, thus generating anisotropy in the rocks’ stress field. The porosity filled with water phase is the structural weakness that is being sensed by strike angle detection near the impact crater boundaries. This is consistent with formation of circular fault systems around the impact craters, where the porosity would form preferentially along the faults formed by impact process^54^. Note, that near the south pole, there is a large region of aligned CF values (labelled “P” in Fig. 4), away from the conspicuously visible major impact craters or topographical relief areas. This “P” region is, however, on the boundary of much larger impact structure, south pole Aitken basin, that significantly modified the early Moon’s crust^55^, and such mega impact event, may have significantly modified the fracture and related pore space network that could be subsequently filled with the water phase, weakening the rocks modified already by impact even further.

The water phase has been shown to exist in the polar regions of the Moon^45,50,56^. Polar water phase has been proposed to come from the Sun^57^. However, most recently it was shown the oxygen ions entering the Moon’s vicinity must source from Earth^58^, when exposed to the magnetotail of Earth^2,4,5,17^. Earth's atmospheric escape would provide the supply of the water molecules to the Moon. The hydrogen cations and oxygen anions are free to react with each other, due to their electronegativity differences when they get close. Then, the chemical bonds re-form to make water molecules while an additional energy is released, which propagates the exothermic reaction further. Earth's atmospheric escape effect^14,26^ serves as a potential source of unaccounted ions^5^ escaping from the Earth into the plasma sheet, and when the Moon passes through this sheet, certain number of oxygen and hydrogen ions is trapped on the Moon. These proposed regions, both in the southern and northern hemispheres, may contain significant subsurface water phase deposits. While these regions were detected from the gravity field aspects, their detection is partially supported from the epithermal neutron emission made from LEND observations onboard LRO, where two out of three Neutron Suppression Regions in this area (NSR S1 and NSR S4) partly overlap with the regions with potentially volumetrically significant water phase detected (Mitrofanov et al., 2012, e.g., their Figure 5)^34^. Detection of porosity through strike angles (Fig. 2) would reach depth of tens of kilometers and would not detect porosity distributed only few meters under the surface, whereas the neutron detection is sensitive only to the very top surface of the Moon (0.5 m) and thereby not detect any water in deeper subsurface. Despite this difference in detection depth, it is remarkable that there is an overlap of these two contrasting water phase detection approaches. At this point, our results coupled with those from prior observation of neutrons suppression regions (NSR) both point to the areas with potential volumes of water phased deposits. While NSR locations are not directly tied to the Lunar impact structures, alignments of strike angles clusters near the rims of large craters and this suggests that strike angle alignment analysis is a straightforward way how to detect remotely significant amounts of water phase on planets.

Our gravity aspect method revealed that strike angles are related to *T*_*zz*_ gravity aspects, the second derivative of vertical gravity disturbance, and sensitive to gravity signature of craters and faulting. However, we note that the gravity field can also be analyzed for horizontal gravity gradients^59^ that can detect potential tabular dike features that are hidden under the surface damaged by impact cratering. This suggests evidence of extensional lithospheric processes at pre-Nectarian to Nectarian age^59^. While our strike angle method was applied in areas very close to the poles (85–90), our vertical T_zz_ gravity aspects was most sensitive to vertical gradients, and we believe our method was not sufficiently sensitive to the linear tabular dikes, for example, detected by Andrews-Hanna et al.^59^. In addition, the tabular dikes mentioned were mostly distributed further from the poles, where our analysis was restricted to 85°–90° from the poles.

## Conclusion

The origin of the water phase on the Moon has not yet been uniquely identified. In this work, we apply recent observations that part of the Earth’s atmosphere may have been transported to the Moon via the novel hydro-magnetospheric-plasma tail and exposing the Moon’s surface with terrestrial H_2_O. We proposed the Moon’s interaction with the geomagnetic tail allows terrestrial ions capture that combine into water molecules and allows water phase deposits on the Moon. Crater impacts, forming structural extensions and fractures, allow suitable pore space networks for hosting large subsurface liquid water reservoirs. Back of envelope calculation suggested several thousands of cubic kilometers of water phase may have accumulated this way into the subsurface of the Moon over the past 3.5 billions of years.

We applied a new method for presenting the gravity aspects signature on the Moon, involving functions derived from gravitational potential, descriptors, and modeling the gravity field. This new method has sensitivity to the structural anisotropy of the Moon’s regolith, especially near impact craters. The gravity aspects method detected specific regions near the north and south poles, which point to the likelihood of significant volume of water phase-filled pore space. It seems likely that identified regions in this work hold significant amounts of water phase, suitable for resource utilization plans of future planned missions (Artemis^1^).

## Supplementary Information

Supplementary Information.
