# Supplementary material for: Distribution of water phase near the poles of the Moon from gravity aspects
Source: Sci Rep. 2022 Mar 16;12:4501. doi: 10.1038/s41598-022-08305-x (PMC8927600; doi:10.1038/s41598-022-08305-x)
Supplement: Supplementary file 1 — Supplementary Information. [file 41598_2022_8305_MOESM1_ESM.docx]

**Distribution of water near the poles of the Moon from gravity aspects**

**Supplementary material**

**Gunther Kletetschka** **^1, 2*^, Jaroslav Klokočník ^3^, Nicholas Hasson ^2, 4^ Jan Kostelecký ^5, 6^, Aleš Bezděk ^3, 7^, and Kurosh Karimi ^1^**

^1^ Institute of Hydrogeology, Engineering Geology and Applied Geophysics, Faculty of Science, Charles University, Prague 12843, Czech Republic.

^2^ Geophysical Institute, University of Alaska - Fairbanks, AK 99709 Fairbanks 903 N Koyukuk Drive, AK, USA

^3^ Astronomical Institute, Czech Academy of Sciences, CZ 251 65 Ondřejov, Fričova 298, Czech Republic

^4^ Water and Environmental Research Center, Institute of Northern Engineering, University of Alaska Fairbanks - Fairbanks, AK 99775 1764 Tanana Loop AK, USA

^5^ Research Institute of Geodesy, Topography and Cartography, CZ 250 66 Zdiby 98, Czech Republic

^6^ Faculty of Mining and Geology, VSB-TU Ostrava, CZ 708 33 Ostrava, Czech Republic

^7^ Faculty of Civil Engineering, Czech Technical University in Prague, CZ 166 29 Praha 6, Czech Republic

^*^ Gunther Kletetschka, gkletetschka@alaska.edu, corresponding author

Jaroslav Klokočník, jklokocn@asu.cas.cz

Jan Kostelecký, kost@fsv.cvut.cz

Aleš Bezděk, bezdek@asu.cas.cz

Nicholas Hasson, nhasson@alaska.edu

Kurosh Karimi, karimik@natur.cuni.cz

*ORCID*:

Gunther Kletetschka: https: //orcid.org/0000-0002-0645-9037

Jaroslav Klokočník: https: //orcid.org/0000-0003-1034-8089

Nicholas Hasson: https://orcid.org/0000-0003-2351-8358

Jan Kostelecký: https://orcid.org/0000-0002-8053-6184

Aleš Bezděk: https: //orcid.org/0000-0003-2790-2664

Kurosh Karimi: https: //orcid.org/0000-0002-3496-8330

Notes to Theory applied to the Moon

We calculate all the gravity aspects including the strike angle *θ* defined as (see ^19,1^ and references therein):

| $\tan2\theta\mathbf{=}2 \frac{T_{xy}\left( T_{xx}+T_{yy} \right)+T_{xz}T_{yz}}{T_{xx}^{2}-T_{yy}^{2}+T_{xz}^{2}-T_{yz}^{2}}=2\frac{{-T}_{xy}T_{zz}+T_{xz}T_{yz}}{T_{xz}^{2}-T_{yz}^{2}+T_{zz}(T_{xx}-T_{yy})}$ | (1) |
| --- | --- |

*T_ij_* are the Marussi tensor’s components ^2^, where the tensor is made of five independent gravity gradients. These components are non-linear combinations made from the harmonic geopotential coefficients from a gravity field model of the Moon and represent the input data for all our analyses. While the strike angle *θ* is the main direction of the Marussi tensor mathematically; in geophysics sense, it indicates stress anisotropy in a specific location. The strike angles tend to be parallel to the schistosity and/or presence of micro-faults. The strike angles are aligned inside and within a certain distance from the impact craters ^3,4^.

Under any coordinate transformation, the Marussi tensor **Γ** preserves three gravity invariant parameters (***I_0_***, ***I_1_***, ***I_2_***):

| $\boldsymbol{I}_{\mathbf{0}}\mathbf{=}trace \left( \boldsymbol{\Gamma} \right)=T_{\mathrm{xx}}+T_{\mathrm{yy}}+T_{\mathrm{zz}}=$ 0 |  |
| --- | --- |
| ***I_1_*** = *(T_xx_T_yy_+T_yy_T_zz_+T_xx_T_zz_) – (T_xy_^2^+T_yz_^2^+T_xz_^2^)* = $\frac{1}{2}\sum_{\left\{ i,j \right\}\in\left\{ x,y,z \right\}} \left( T_{ii}T_{jj}-T_{ij}^{2} \right)$ |  |
| ***I_2_*** = *det (****Г****) = (T_xx_(T_yy_T_zz_-T_yz_^2^) + T_xy_(T_yz_T_xz_ – T_xy_T_zz_) + T_xz_ (T_xy_T_yz_-T_xz_T_yy_).* |  |

Since **Γ** is a symmetric matrix, its eigenvalues are real, and eigenvectors are perpendicular. From the reality of the eigenvalues, *I* follows to be between 0 and 1 ^2^.

| $0\boldsymbol{\leq}I\mathbf{=-}\frac{\left( I_{2}/2 \right)^{2}}{\left( I_{1}/3 \right)^{3}}\boldsymbol{\leq}1$ | (2) |
| --- | --- |

When the causative body is nearly 2D, *I* approaches zero; with the higher *I*, we detect more 3D morphology of the causative object. For example, when detecting oil and gas deposits, we have more 2D like I values. The gravity aspects indicate only geologic structures with density contrasts, like anticlines, faults, salt domes, which may occasionally contain concentrations of aligned strike angles not directly due to gas, oil or water but due to density anisotropy. Correlation does not imply causation and therefore when our data and method are supportive for the existence of the water deposits on the Moon, it does not confirm its existence*.*

The strike angles *θ* are normally oriented chaotically. Sometimes they are aligned or form a halo around the impact craters. The spatial distribution of the aligned strike angles may become a new and accessible empirical geophysical tool for the recognition of potential water deposits within the Moon’s regolith. Such combed strike angles (specifically oriented) have already been investigated for the Earth (to detect the anisotropy due to various origin) ^3-5^. We have developed an empirical statistical Comb Factor (CF)*.* The CF expresses a degree of alignment of the strike angles. The *θ* angles were expressed as horizontal unit vectors. We use a scalar product of the group of neighboring unit vectors (twenty-five adjacent data points). Thus, when the cosine of the angle of the neighboring vectors approaches 1 (~0-degree angle between the unit vectors), alignment is maximum. However, when the scalar product of these vectors is close to zero, the neighboring vectors are misaligned to maximum degree, close to perpendicular to each other. Thus, the CF is calculated like this: For the point where we wish to express the alignment, we choose a constant area containing its closest twenty-four neighbors. For these vectors contained in this area we express an arithmetic mean of all the scalar products between the vector for which we calculate alignment and its neighboring vectors. The result of this application allows to map out areas with chaotic distribution of theta angles (CF ~ 0) and areas with nearly perfect alignment, (CF > 0.99).

Following are figures illustrating the strike angles around the north and south poles of the Moon. These figures illustrate a robustness of this approach in relation to detection of alignment of this parameter in respect to the potential detection of the permafrost deposits in the polar regions of the Moon (see the main text for more detailed relation).


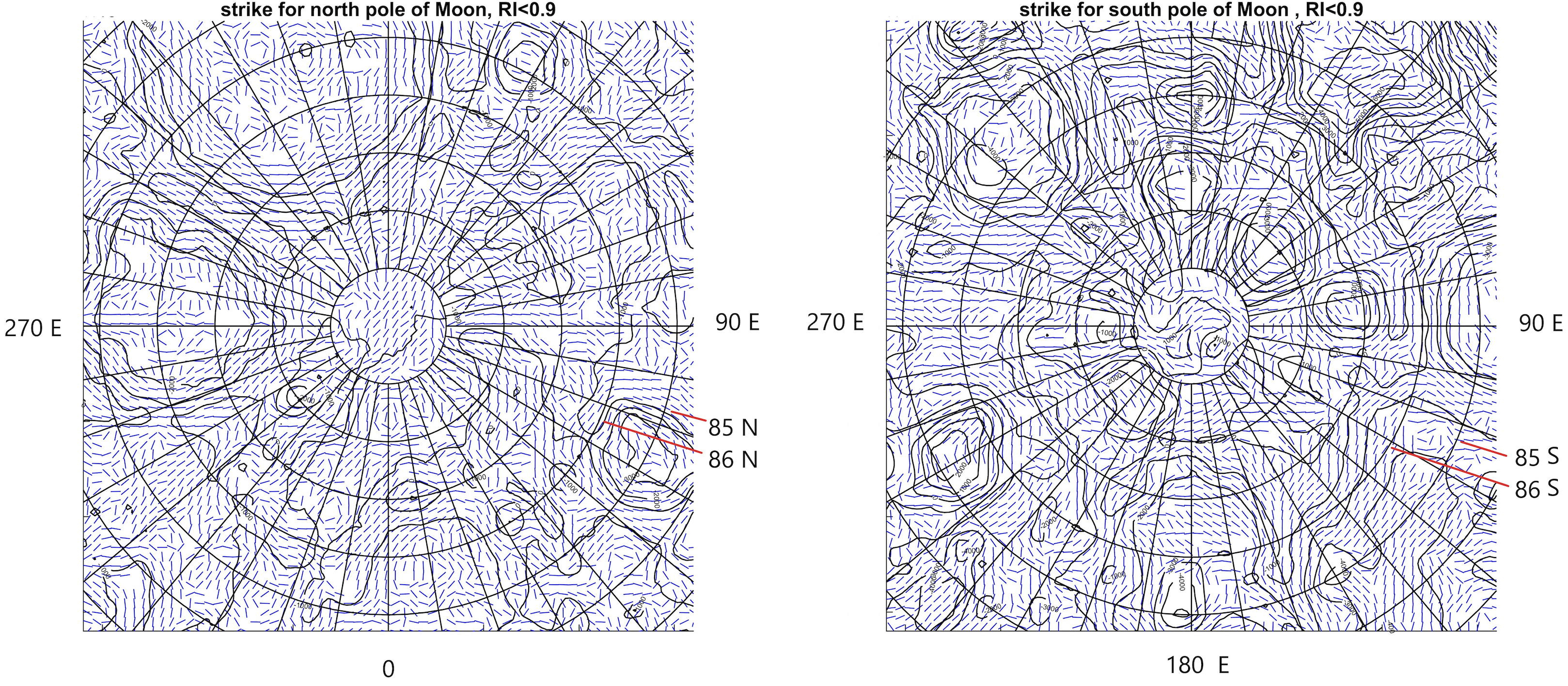


Figure S1: Topography and plot of strike angles for ratio *I*<0.9 (see equation 2), sensitive to weakness directions of the rocks in subsurface structures near the north and south poles of the Moon. Data were plotted by MATLAB.


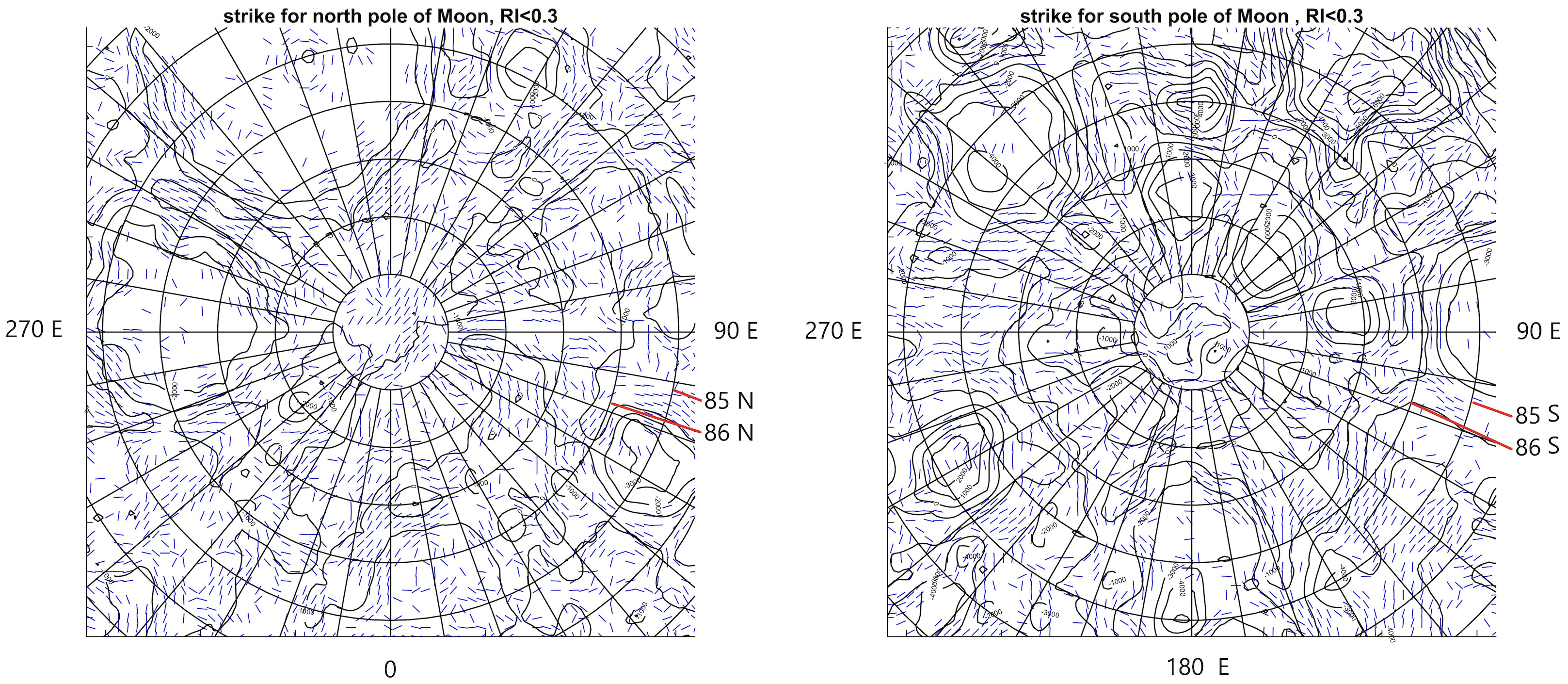


Figure S2: Topography and plot of strike angles for ratio *I*<0.3 (see equation 2), sensitive to weakness directions of the rocks in subsurface structures near the north and south poles of the Moon. Data were plotted by MATLAB.


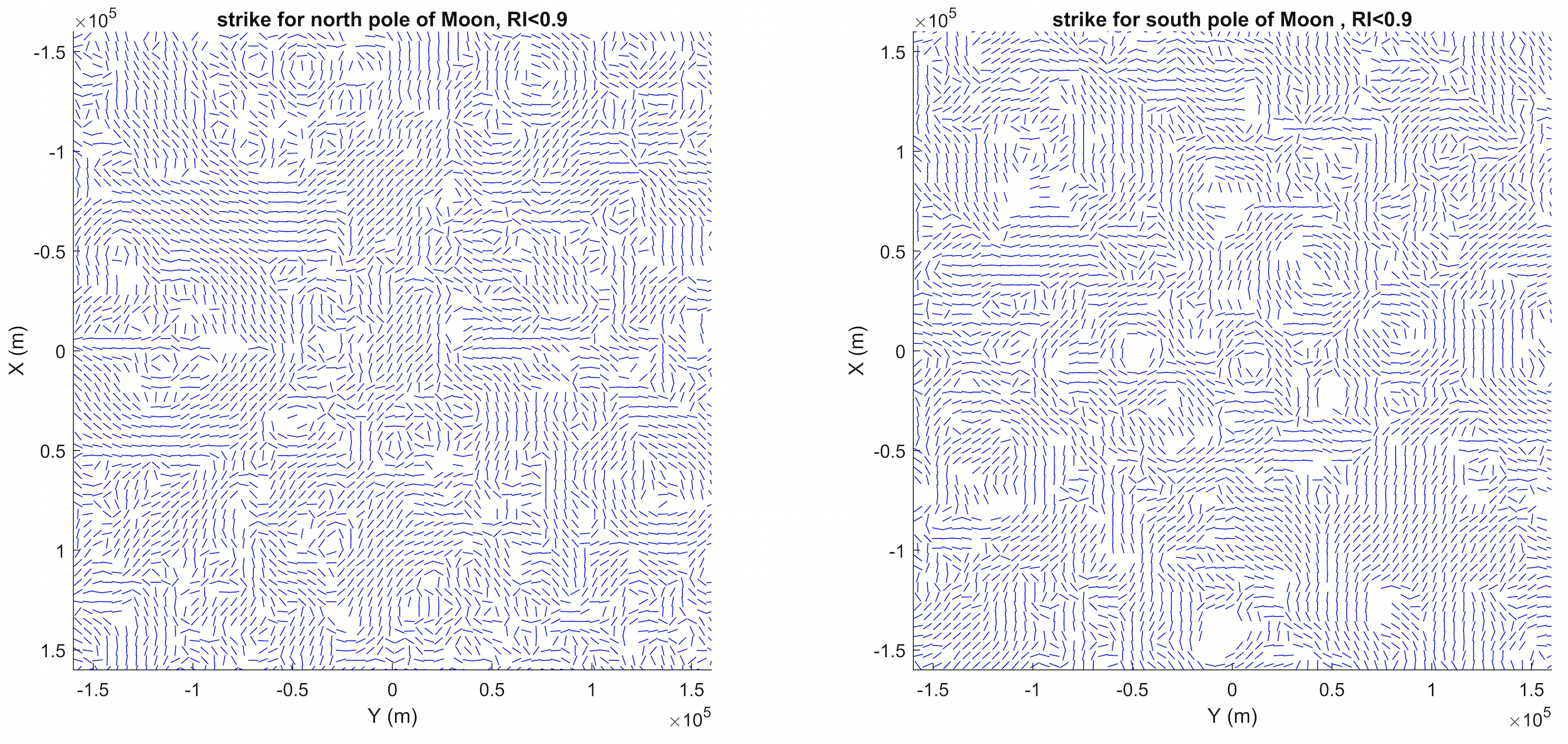


Figure S3: Same areas as in Figure S1, plot of strike angles for ratio *I*<0.9 (see equation 2), sensitive to weakness directions of the rocks in subsurface structures near the north and south poles of the Moon. Dimensions are in meters. Topography and latitude longitude lines were omitted for clarity. Data were plotted by MATLAB.


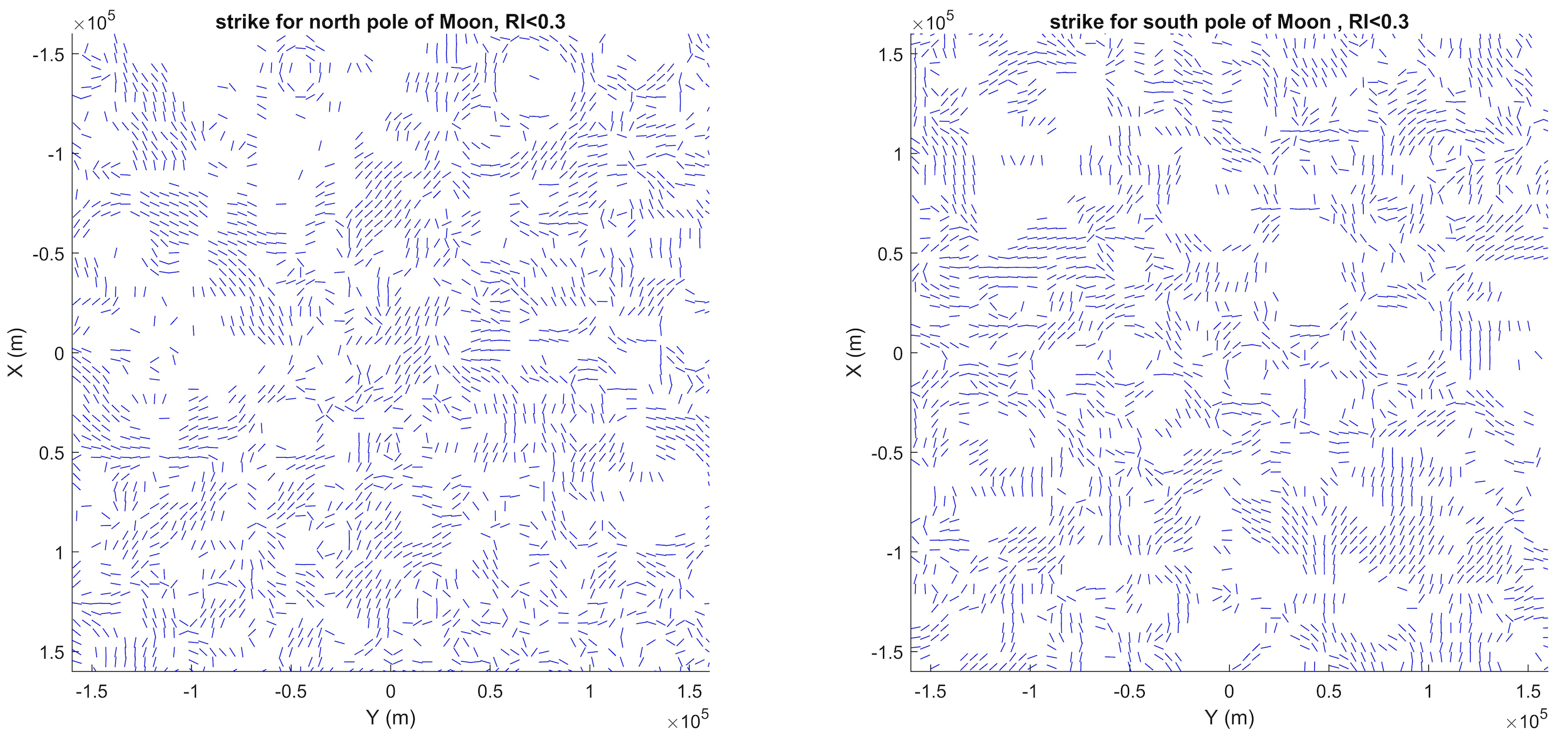


Figure S4: Same areas as in Figure S2, plot of strike angles for ratio *I*<0.3 (see equation 2), sensitive to weakness directions of the rocks in subsurface structures near the north and south poles of the Moon. Dimensions are in meters. Topography and latitude longitude lines were omitted for clarity. Data were plotted by Matlab.


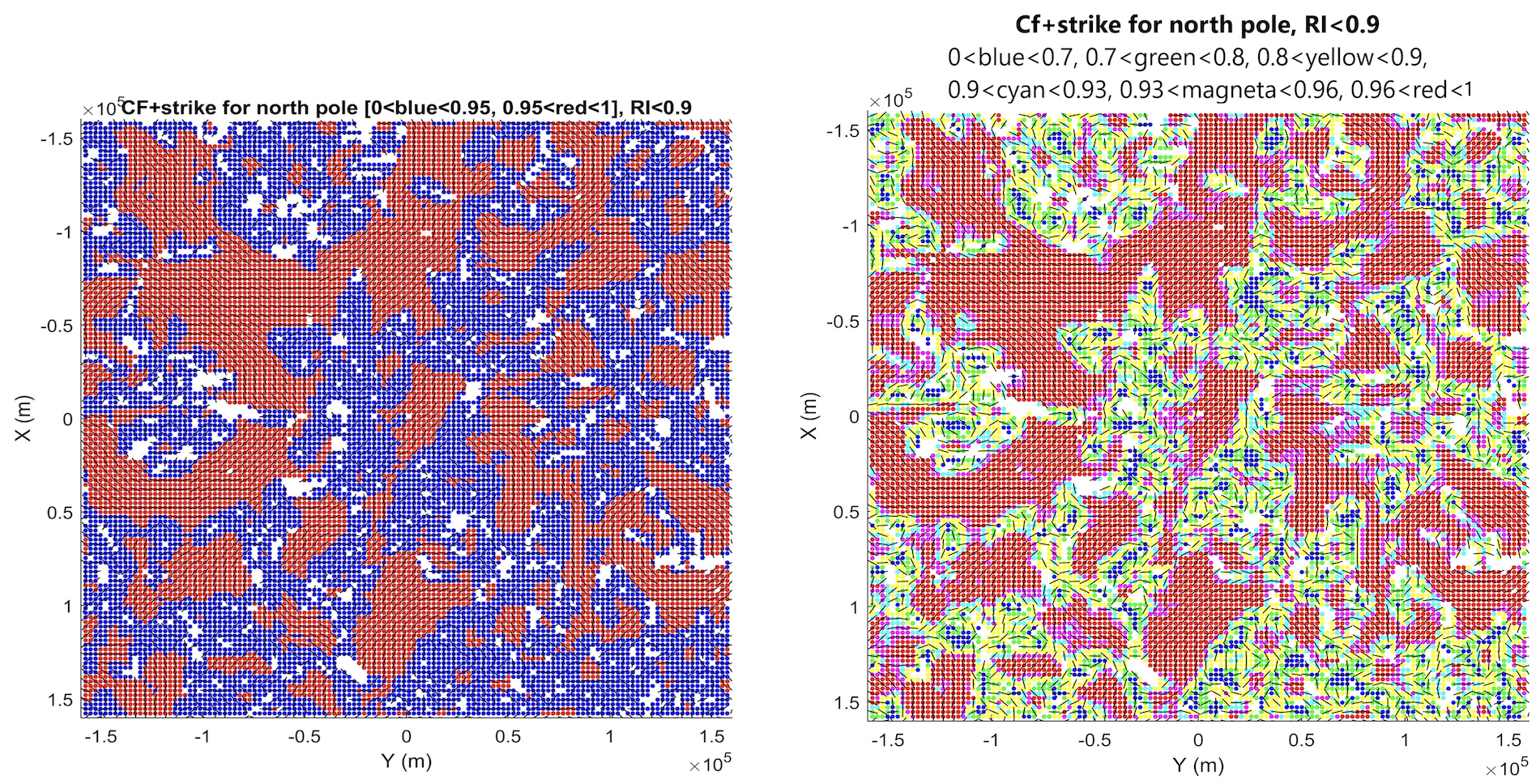


Figure S5: Stability of the comb parameter within the area of north pole of the Moon. Dimensions are in meters. Left panel corresponds to the strike angle plot for the north pole in Figures S1, S3 where the comb factor is between 0.95 and 1.00 is in red color while the rest is in blue. Right panel shows various color scheme identified in the legend. All plots are strike angles for ratio *I*<0.9 (see equation 2), sensitive to weakness directions of the rocks in subsurface structures near the north pole of the Moon. Topography and latitude longitude lines were omitted for clarity. Data were plotted by MATLAB.


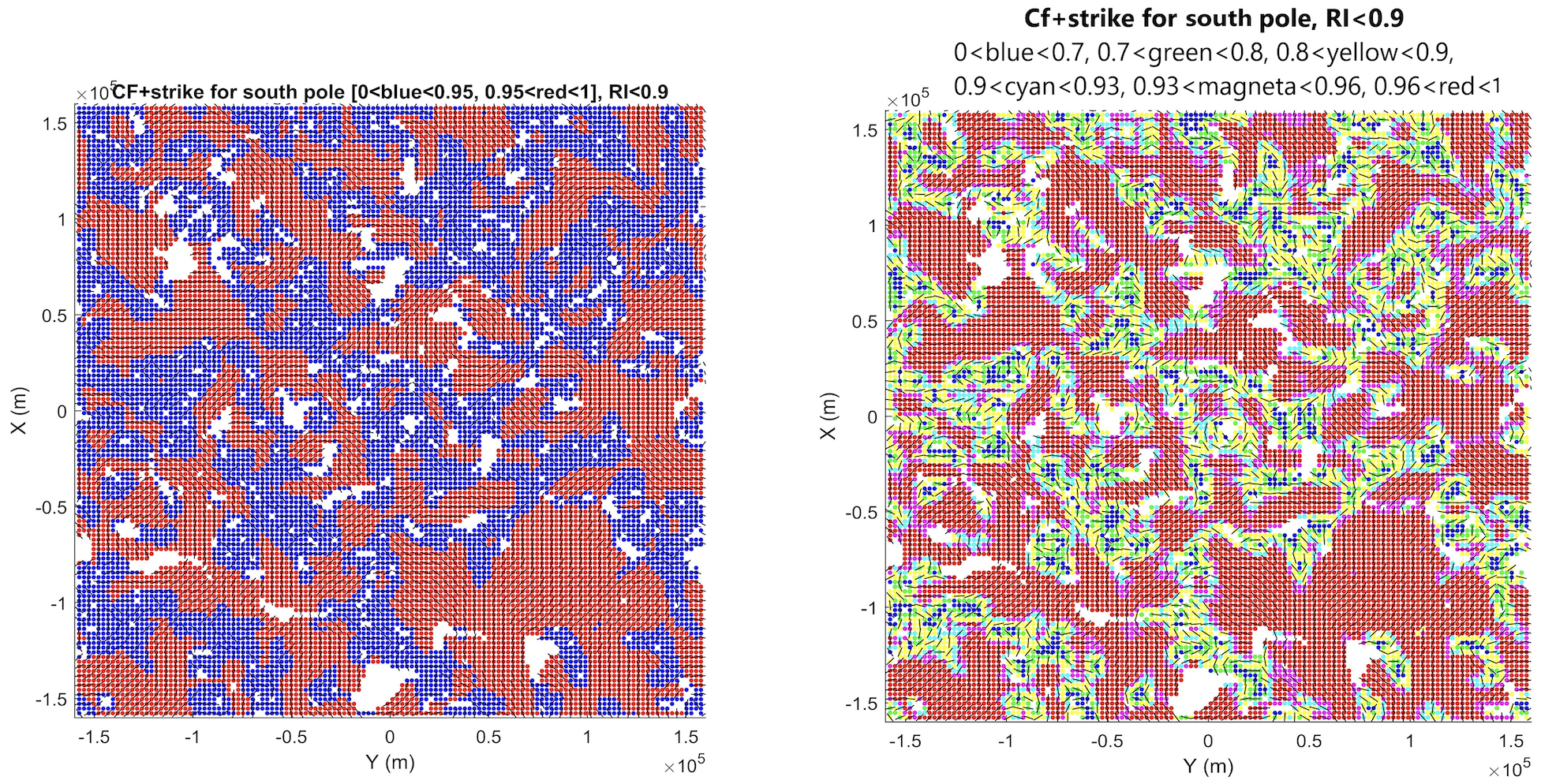


Figure S6: Stability of the comb parameter within the area of south pole of the Moon. Dimensions are in meters. Left panel corresponds to the strike angle plot for the south pole in Figures S2, S4 where the comb factor is between 0.95 and 1.00 is in red color while the rest is in blue. Right panel shows various color scheme identified in the legend. All plots are strike angles for ratio *I*<0.9 (see equation 2), sensitive to weakness directions of the rocks in subsurface structures near the north pole of the Moon. Topography and latitude longitude lines were omitted for clarity. Data were plotted by MATLAB.


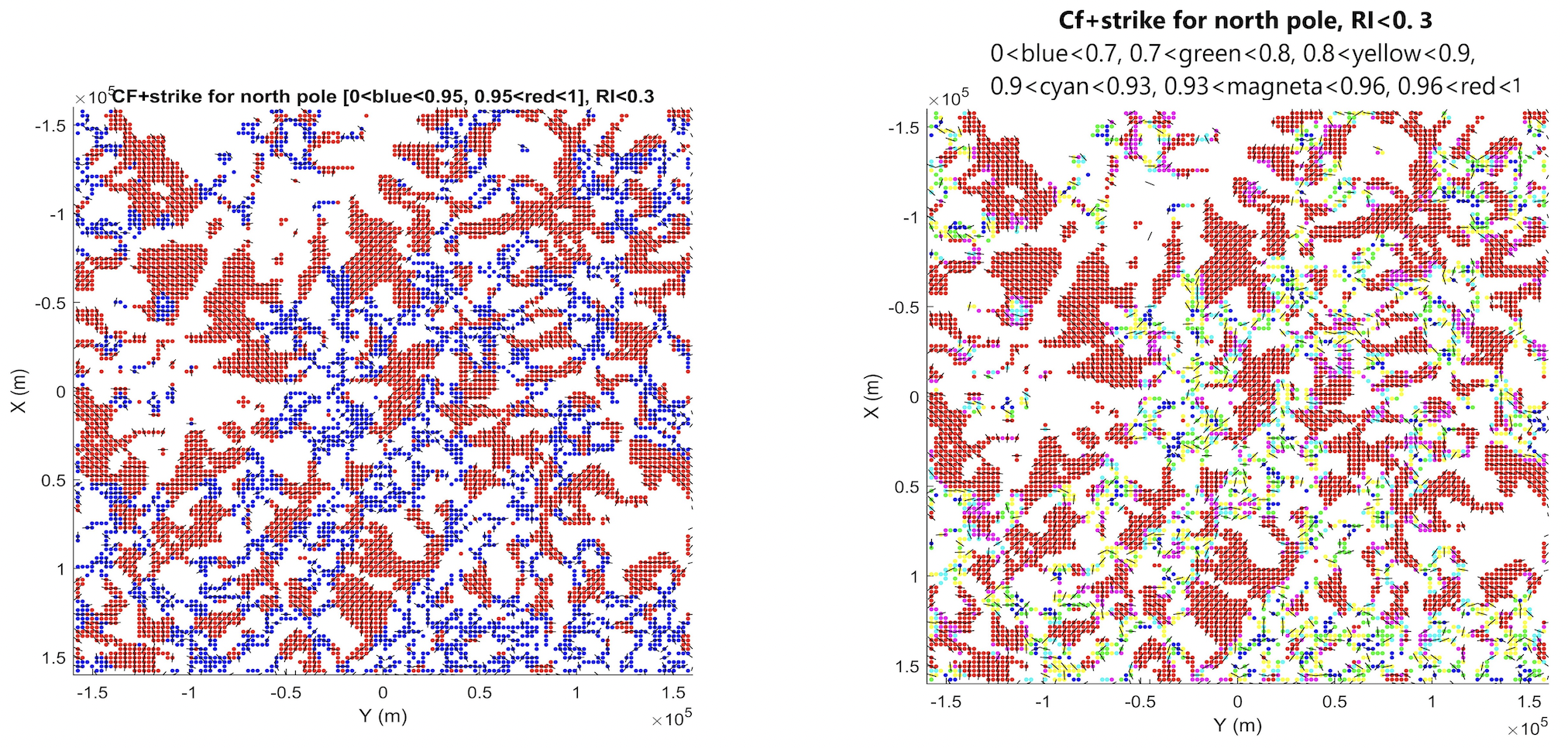


Figure S7: Stability of the comb parameter within the area of north pole of the Moon. Dimensions are in meters. Left panel corresponds to the strike angle plot for the north pole in Figures S1, S3 where the comb factor is between 0.95 and 1.00 is in red color while the rest is in blue. Right panel shows various color scheme identified in the legend. All plots are strike angles for ratio *I*<0.3 (see equation 2), sensitive to weakness directions of the rocks in subsurface structures near the north pole of the Moon. Topography and latitude longitude lines were omitted for clarity. Data were plotted by MATLAB.


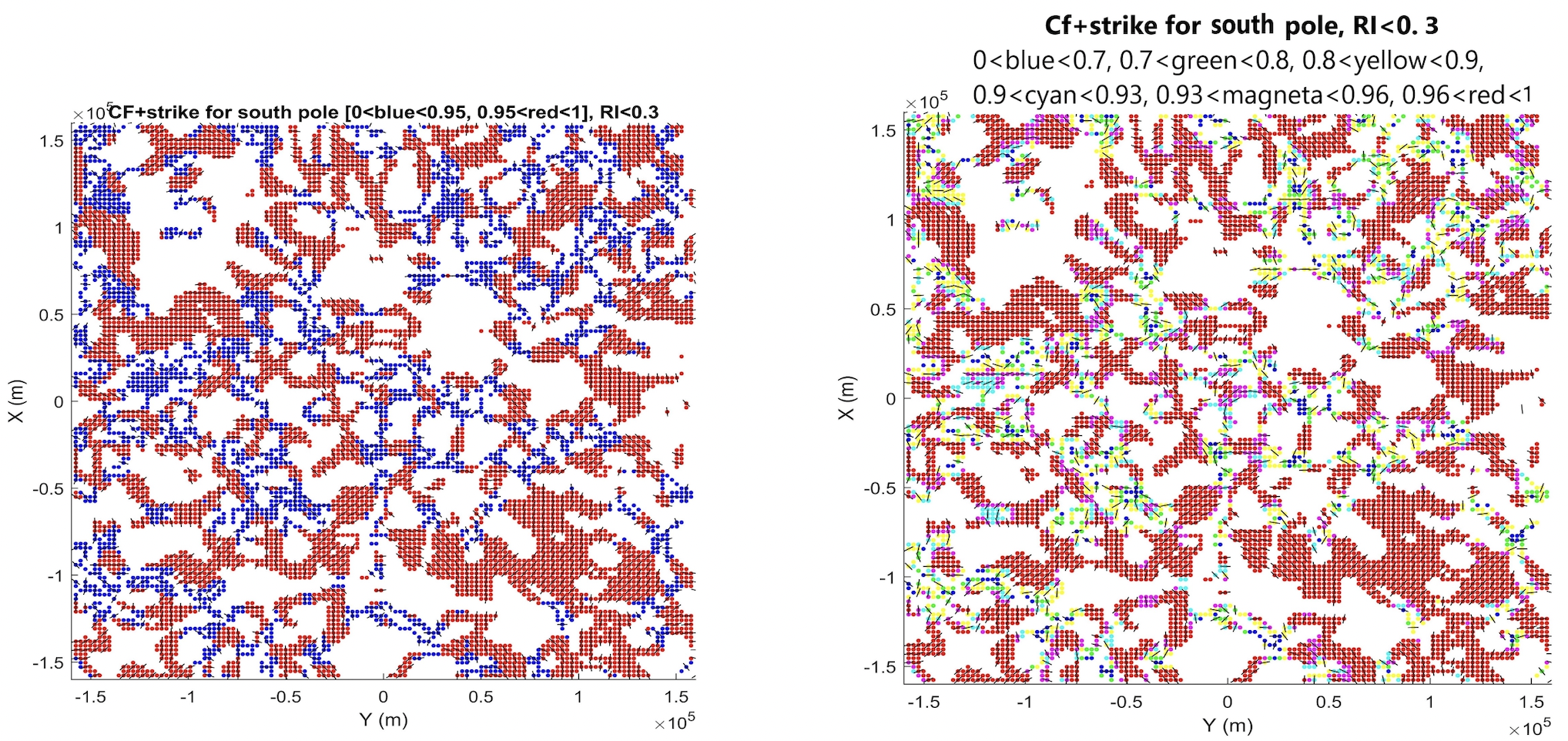


Figure S8: Stability of the comb parameter within the area of south pole of the Moon. Dimensions are in meters. Left panel corresponds to the strike angle plot for the south pole in Figures S2, S4 where the comb factor is between 0.95 and 1.00 is in red color while the rest is in blue. Right panel shows various color scheme identified in the legend. All plots are strike angles for ratio *I*<0.3 (see equation 2), sensitive to weakness directions of the rocks in subsurface structures near the north pole of the Moon. Topography and latitude longitude lines were omitted for clarity. Data were plotted by MATLAB
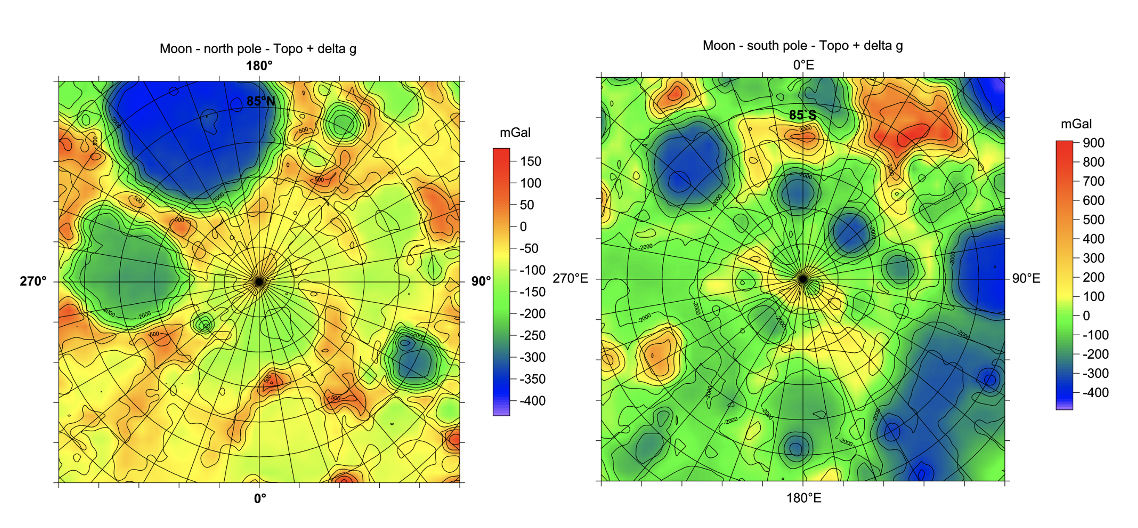


Figure S9: Topography and the gravity disturbance *∆g* [mGal], sensitive to deeper subsurface structures near the north and south poles of the Moon. Data were plotted by Surfer 7.0.


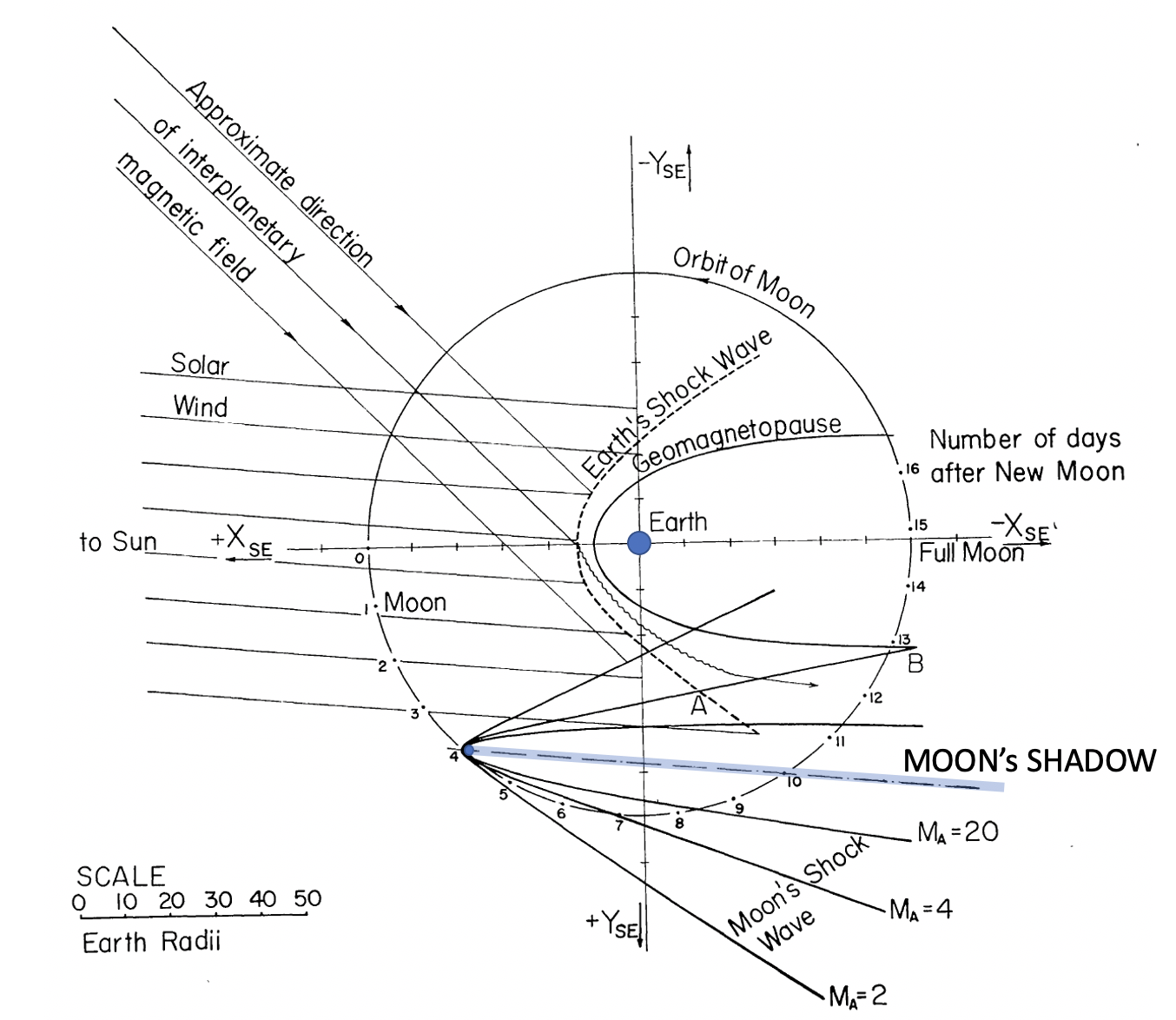


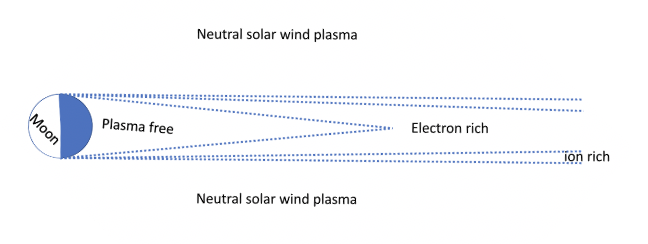


Figure S10: Ecliptic plane topology showing lunar orbit along with its modelled shock waves of different values of the Alfven-Mach number M_A_ when Moon positioned 4 days after the new Moon. The figure also depicts the shadow behind the Moon that may contribute to electrostatic potential disruptions when entering the geomagnetic field (modified from Fig 3, Schneider, 1967 ^6^). Detail shows preferential diffusion of the electrons from the Solar wind plasma into the shadow of the Moon, leaving behind positive ions.

1 Klokočník, J., Kostelecký, J., Cílek, V., Bezděk, A. & Pešek, I. A support for the existence of paleolakes and paleorivers buried under Saharan sand by means of "gravitational signal" from EIGEN 6C4. *Arab. J. Geosci.* **10**, 28, doi:10.1007/s12517-017-2962-8 (2017).

2 Pedersen, L. B. & Rasmussen, T. M. THE GRADIENT TENSOR OF POTENTIAL-FIELD ANOMALIES - SOME IMPLICATIONS ON DATA-COLLECTION AND DATA-PROCESSING OF MAPS. *Geophysics* **55**, 1558-1566, doi:10.1190/1.1442807 (1990).

3 Klokočník, J., Kostelecký, J., Bezděk, A. & Kletetschka, G. Gravity strike angles: a modern approach and tool to estimate the direction of impactors of meteoritic craters. *Planetary and Space Science*, 105113, doi:https://doi.org/10.1016/j.pss.2020.105113 (2020).

4 Klokočník, J. *et al.* Support for two subglacial impact craters in northwest Greenland from Earth gravity model EIGEN 6C4 and other data. *Tectonophysics* **780**, 228396, doi:https://doi.org/10.1016/j.tecto.2020.228396 (2020).

5 Klokočník, J., Kostelecký, J. & Cílek, V. *Subglacial and underground structures detected from recent gravito-topography data*. (Cambridge Scholars Publishing, 2020).

6 Schneider, O. INTERACTION OF MOON WITH EARTHS MAGNETOSPHERE. *Space Sci. Rev.* **6**, 655-+ (1967).
